# Transformations of superpositions by means of incoherent operations

**DOI:** 10.1038/s41598-020-63661-w

**Published:** 2020-05-19

**Authors:** Marcelo Losada, Gustavo M. Bosyk, Hector Freytes, Giuseppe Sergioli

**Affiliations:** 1 Università degli Studi di Cagliari, Via Is Mirrionis 1, I-09123 Cagliari, Italy; 2 Instituto de Física La Plata, UNLP, CONICET, Facultad de Ciencias Exactas, La Plata, Argentina

**Keywords:** Quantum information, Quantum mechanics

## Abstract

In this paper we study how the coherence of a superposition of pure states is related with the coherence of its components. We consider two pure initial states and two pure final coherent states, such that the former ones cannot be transformed into the latter ones by means of incoherent transformations. In this situation, we analyze conditions for the existence of superpositions of the initial states that can be transformed into superpositions of the final states. In particular, we consider superpositions formed by quantum states belonging to orthogonal subspaces. By appealing to the majorization theory, we obtain necessary and sufficient conditions for such transformations to be possible. Finally, we provide some examples that illustrate the difference between the obtained conditions and the necessary criterion based on the relative entropy of coherence.

## Introduction

Quantum coherence is a fundamental notion of quantum mechanics, being not just a side result of the superposition principle but arguably one of its most fundamental concepts, with outstanding practical relevance. In fact, quantum coherence has recently been identified as a quantum resource^1–3^, and it was reformulated using the general framework of quantum resource theories (see, *e*.*g*., ref. ^3^ for a comprehensive review about the different resource theories of quantum coherence and its applications).

In this work, we are interested in deterministic and exact transformations between coherent pure states by means of incoherent operations. An incoherent operation (IO) is defined as a trace preserving map that admits a Kraus representation ${\{{K}_{n}\}}_{n\in I}$ (with $I$ a finite index set), such that ${\sum }_{n\in I}\,{K}_{n}^{\dagger }{K}_{n}={\mathbb{I}}$ and ${K}_{n}\rho {K}_{n}^{\dagger }/{\rm{Tr}}[{K}_{n}\rho {K}_{n}^{\dagger }]\in  {\mathcal I}$ for all $n\in I$ and $\rho \in  {\mathcal I}$, where ${\mathcal{I}}=\{\rho :\rho ={\sum }_{i=0}^{m-1}\,{p}_{i}|i\rangle \langle i|,{\rm{w}}{\rm{i}}{\rm{t}}{\rm{h}}\,\,\,{p}_{i}\ge 0\,\,\,{\rm{a}}{\rm{n}}{\rm{d}}\,\,\,{\sum }_{i=0}^{m-1}\,{p}_{i}=1\}$ is the set of incoherent states and $\{|i\rangle {\}}_{i=0}^{m-1}$ is the incoherent basis^1^. In this way, incoherent operations map incoherent states into incoherent states. We will use the notation $|\Psi \rangle \mathop{\to }\limits_{{\rm{I}}{\rm{O}}}|\Phi \rangle$ to mean that the state |Ψ〉 can be transformed into the state |Φ〉 using an incoherent operation.

It has been shown that the necessary and sufficient conditions for the transformation $|\Psi \rangle \mathop{\to }\limits_{{\rm{IO}}}|\Phi \rangle$ to be possible are given by the majorization relation (see refs. ^4–7^). More specifically, let us consider an $m$-dimensional Hilbert space with the incoherent basis given by $\{|i\rangle {\}}_{i=0}^{m-1}$. Let $|\Psi \rangle ={\sum }_{i=0}^{m-1}\,{\psi }_{i}|i\rangle$ and $|\Phi \rangle ={\sum }_{i=0}^{m-1}\,{\phi }_{i}|i\rangle$ be two pure states, where the coefficients ${\{{\psi }_{i}\}}_{0\le i\le m-1}$ and ${\{{\phi }_{i}\}}_{0\le i\le m-1}$ are complex numbers satisfying ${\sum }_{i=0}^{m-1}|{\psi }_{i}{|}^{2}={\sum }_{i=0}^{m-1}|{\phi }_{i}{|}^{2}=1$. Let $p(\Psi )$ and $p(\Phi )$ be the probability vectors associated with these pure states in the incoherent basis, i.e., ${p}_{i}(\Psi )=|{\psi }_{i}{|}^{2}$ and ${p}_{i}(\Phi )=|{\phi }_{i}{|}^{2}$. Then (see refs. ^4–7^),

1 $$|\Psi \rangle \mathop{\to }\limits_{{\rm{I}}{\rm{O}}}|\Phi \rangle \,\,\,{\rm{i}}{\rm{f}}\,{\rm{a}}{\rm{n}}{\rm{d}}\,{\rm{o}}{\rm{n}}{\rm{l}}{\rm{y}}\,{\rm{i}}{\rm{f}}\,\,\,p(\Phi )\succcurlyeq p(\Psi ),$$

where $p(\Phi )\succcurlyeq p(\Psi )$ reads as $p(\Phi )$ majorizes $p(\Psi )$ and means that ${\sum }_{i=0}^{k}\,{p}_{i}^{\downarrow }(\Phi )\ge {\sum }_{i=0}^{k}\,{p}_{i}^{\downarrow }(\Psi )$ for all $k\in \{0,\ldots ,m-1\}$, with the symbol ^↓^ indicating that the components of the probability vectors are sorted in a decreasing order (see, *e*.*g*., ref. ^8^ for an introduction to majorization theory and refs. ^9,10^ for a comprehensive review about its applications on quantum information). Notice that relation (1) can be seen as the analogous of the celebrated Nielsen’s theorem^11^ for quantum coherence.

Since coherence is a consequence of the superposition principle, it is important to understand how the coherence of a superposition of coherent states is related with the coherence of the superposed states. Some progress has been made in this direction. For example, for a given superposition of two coherent states $|a\rangle$ and $|b\rangle$ of the form $|\Psi \rangle ={\alpha }_{1}|a\rangle +{\alpha }_{2}|b\rangle$, it has been recently investigated the relation among the coherence of the superposition $C(|\Psi \rangle )$ and the coherence of the superposed states $C(|a\rangle )$ and $C(|b\rangle )$^12^. In particular, lower and upper bounds of $C(|\Psi \rangle )$ in terms of $C(|a\rangle )$ and $C(|b\rangle )$ for several measures of quantum coherence, like the relative entropy of coherence and the ${\ell }_{1}$-norm of coherence, have been recently obtained in refs. ^12–14^.

However, this relationship is not yet fully characterized. Therefore, in this work we provide a step forward in the characterization of the relationship between the coherence of a superposition and the coherence of the superposed states. In particular, we consider the following scenario. Let $|a\rangle$, $|b\rangle$ be two initial pure states, and $|c\rangle$, $|d\rangle$ be two final coherent states, such that it is not possible to transform any of the initial states into the final ones, i.e.,

2 $$|a\rangle \mathop{ \nrightarrow }\limits_{{\rm{I}}{\rm{O}}}|c\rangle \,\,\,{\rm{a}}{\rm{n}}{\rm{d}}\,\,\,|a\rangle \mathop{ \nrightarrow }\limits_{{\rm{I}}{\rm{O}}}|d\rangle ,$$

3 $$|b\rangle \mathop{ \nrightarrow }\limits_{{\rm{I}}{\rm{O}}}|c\rangle \,\,\,{\rm{a}}{\rm{n}}{\rm{d}}\,\,\,|b\rangle \mathop{ \nrightarrow }\limits_{{\rm{I}}{\rm{O}}}|d\rangle .$$

In this situation, we aim to find an initial superposition $|\Psi \rangle ={\alpha }_{1}|a\rangle +{\alpha }_{2}|b\rangle$ and a final superposition $|\Phi \rangle ={\beta }_{1}|c\rangle +{\beta }_{2}|d\rangle$ such that the transformation $|\Psi \rangle \mathop{\to }\limits_{{\rm{IO}}}|\Phi \rangle$ is possible. For some particular cases, we will provide necessary and sufficient conditions in terms of the initial and final states $|a\rangle$, $|b\rangle$, $|c\rangle$ and $|d\rangle$ in order to make possible the transformation under study.

## Results

Let us consider a Hilbert space ${\mathcal H}$ of dimension $m$, and an incoherent basis ${\{|i\rangle \}}_{0\le i\le m-1}$. Let $|a\rangle$, $|b\rangle$, $|c\rangle$, $|d\rangle \in  {\mathcal H}$ be quantum states satisfying the conditions (2) and (3), and let $|\Psi \rangle ={\alpha }_{1}|a\rangle +{\alpha }_{2}|b\rangle$ and $|\Phi \rangle ={\beta }_{1}|c\rangle +{\beta }_{2}|d\rangle$ (with $|{\alpha }_{1}{|}^{2}+|{\alpha }_{2}{|}^{2}=1$, $|{\beta }_{1}{|}^{2}+|{\beta }_{2}{|}^{2}=1$ and ${\alpha }_{1},{\alpha }_{2},{\beta }_{1},{\beta }_{2}\ne 0$) be two arbitrary superpositions. We want to characterize the values of ${\alpha }_{1}$, ${\alpha }_{2}$, ${\beta }_{1}$ and ${\beta }_{2}$ for which the transformation $|\Psi \rangle \mathop{\to }\limits_{{\rm{IO}}}|\Phi \rangle$ is possible. Let $p(\Psi )$ and $p(\Phi )$ be the probability vectors associated with the superpositions |Ψ〉 and |Φ〉 in the incoherent basis, respectively. According to relation (1), the transformation $|\Psi \rangle \mathop{\to }\limits_{{\rm{IO}}}|\Phi \rangle$ is possible if and only if the probability vectors $p(\Psi )$ and $p(\Phi )$ satisfy the majorization relation, i.e., $p(\Phi )\,\succcurlyeq \,p(\Psi )$.

Let us confine our problem to the case of superpositions of quantum states that belong to orthogonal subspaces. This case is particularly interesting because quantum states from orthogonal subspaces play an important role in quantum information and encoding^15^. More precisely, we consider $|a\rangle ={\sum }_{i=0}^{m-1}\,{a}_{i}|i\rangle$, $|b\rangle ={\sum }_{i=0}^{m-1}\,{b}_{i}|i\rangle$, $|c\rangle ={\sum }_{i=0}^{m-1}\,{c}_{i}|i\rangle$, $|d\rangle ={\sum }_{i=0}^{m-1}\,{d}_{i}|i\rangle$, with ${a}_{i}{b}_{i}=0$ and ${c}_{i}{d}_{i}=0$, for all $0\le i\le m-1$. Notice that this situation was considered when studying the coherence of superpositions in ref. ^12^, and also in the case of entanglement of superposition of bipartite systems in refs. ^16,17^. As we are interested in the probability vectors $p(\Psi )$ and $p(\Phi )$, and the states $|a\rangle$, $|b\rangle$ and $|c\rangle$, $|d\rangle$ belong to orthogonal subspaces, we can assume without loss of generality that all coefficients are positive or zero, i.e., ${a}_{i},{b}_{i},{c}_{i},{d}_{i}\ge 0$. For the same reason, we can also assume that ${\alpha }_{1}=\sqrt{\alpha }$, ${\alpha }_{2}=\sqrt{1-\alpha }$, and ${\beta }_{1}=\sqrt{\beta }$, ${\beta }_{2}=\sqrt{1-\beta }$, therefore the initial and final superpositions have the form

4 $$|\Psi \rangle =\sqrt{\alpha }|a\rangle +\sqrt{(1-\alpha )}|b\rangle ,\,\,\,|\Phi \rangle =\sqrt{\beta }|c\rangle +\sqrt{1-\beta }|d\rangle .$$

Under these constrains, the first non-trivial case for which there are pure states satisfying (2) and (3) appears when both states |*c*〉 and |*d*〉 have two non-null coefficients in the incoherent basis. Because, otherwise, |*c*〉 and |*d*〉 are incoherent states and conditions (2) and (3) cannot be satisfied. For this to be possible the dimension of the Hilbert space has to be more than 3. We are going to restrict our analysis to this first case, i.e., we consider a Hilbert space of dimension equal to 4. For the states |*a*〉 and |*b*〉 there are only two possible situations: (1) they have three and one non-null coefficients in the incoherent basis, or (2) they have two non-null coefficients in the incoherent basis.

In case (1), we can assume without loss of generality that $|a\rangle =|0\rangle$ and $|b\rangle =\sqrt{{b}_{1}}|1\rangle +\sqrt{{b}_{2}}|2\rangle +\sqrt{{b}_{3}}|3\rangle$. Moreover, we can choose $|c\rangle =\sqrt{{c}_{0}}|0\rangle +\sqrt{{c}_{1}}|1\rangle$ and $|d\rangle =\sqrt{{d}_{2}}|2\rangle +\sqrt{{d}_{3}}|3\rangle$. Let us denote this case as $\mathrm{(1,}\,\mathrm{3)}\mathop{\to }\limits_{{\rm{IO}}}\mathrm{(2,}\,\mathrm{2)}$. In case (2), we can assume that $|a\rangle =\sqrt{{a}_{0}}|0\rangle +\sqrt{{a}_{1}}|1\rangle$ and $|b\rangle =\sqrt{{b}_{2}}|2\rangle +\sqrt{{b}_{3}}|3\rangle$. The states |*c*〉 and |*d*〉 have two possible options: (2.i) $|c\rangle =\sqrt{{c}_{0}}|0\rangle +\sqrt{{c}_{1}}|1\rangle$ and $|d\rangle =\sqrt{{d}_{2}}|2\rangle +\sqrt{{d}_{3}}|3\rangle$, and (2.ii) $|c\rangle =\sqrt{{c}_{0}}|0\rangle +\sqrt{{c}_{2}}|2\rangle$ and $|d\rangle =\sqrt{{d}_{1}}|1\rangle +\sqrt{{d}_{3}}|3\rangle$. However, the case (2.ii) can be transformed into the case (2.i) by means of the simple incoherent operation $|1\rangle \mathop{\leftrightarrow }\limits_{{\rm{IO}}}|2\rangle$. Therefore, we only have to consider the case (2.i). Let us denote this case as $(2,2)\mathop{\to }\limits_{{\rm{IO}}}(2,2)$.

For these two cases we will obtain necessary and sufficient conditions for coefficients $\alpha$ and $\beta$ in order to allow the transformation under study.

**Case:** $$(1,3)\mathop{\to }\limits_{{\rm{IO}}}(2,2)$$

In this case, the pure states $|a\rangle$, $|b\rangle$, $|c\rangle$, $|d\rangle$ have the following form

5 $$|a\rangle =|0\rangle ,$$

6 $$|b\rangle =\sqrt{{b}_{1}}|1\rangle +\sqrt{{b}_{2}}|2\rangle +\sqrt{1-{b}_{1}-{b}_{2}}|3\rangle ,$$

7 $$|c\rangle =\sqrt{c}|0\rangle +\sqrt{1-c}|1\rangle ,$$

8 $$|d\rangle =\sqrt{d}|2\rangle +\sqrt{1-d}|3\rangle ,$$

with ${b}_{1}\ge {b}_{2}\ge 1-{b}_{1}-{b}_{2} > 0$, $c\ge 1/2$, $d\ge 1/2$ and $c\ge d$ (i.e., $c\ge d\ge 1-d\ge 1-c$). Notice that we can sort the coefficients in this way without loss of generality, since if this is not the case, we can perform a simple permutation (which is an incoherent operation) in order to obtain the expected order. For instance, let us assume that $c\le d$, then applying the permutations $|0\rangle \leftrightarrow |2\rangle$ and $|1\rangle \leftrightarrow |3\rangle$, one obtains $|c\rangle \mathop{\leftrightarrow }\limits_{{\rm{I}}{\rm{O}}}|{d}^{{\rm{{\prime} }}}\rangle =\sqrt{c}|2\rangle +\sqrt{1-c}|3\rangle$ and $|d\rangle \mathop{\leftrightarrow }\limits_{{\rm{I}}{\rm{O}}}|{c}^{{\rm{{\prime} }}}\rangle =\sqrt{d}|0\rangle +\sqrt{1-d}|1\rangle$.

Clearly, since the state |*a*〉 is an incoherent state, it satisfies the condition (2). On the other hand, the coherent state |*b*〉 satisfies the condition (3) if and only if ${b}_{1} > c$. Therefore, the coefficients have to satisfy the following inequalities

9 $${b}_{1} > c\ge d\ge 1-d\ge 1-c > {b}_{2}\ge 1-{b}_{1}-{b}_{2} > 0.$$

In this case, the initial and final superpositions have the following form

10 $$|\Psi \rangle =\sqrt{\alpha }|0\rangle +\sqrt{(1-\alpha ){b}_{1}}|1\rangle +\sqrt{(1-\alpha ){b}_{2}}|2\rangle +\sqrt{(1-\alpha )(1-{b}_{1}-{b}_{2})}|3\rangle ,$$

and

11 $$|\Phi \rangle =\sqrt{\beta c}|0\rangle +\sqrt{\beta (1-c)}|1\rangle +\sqrt{(1-\beta )d}|2\rangle +\sqrt{(1-\beta )(1-d)}|3\rangle .$$

Therefore, the probability vectors in the incoherent basis associated with the superpositions |Ψ〉 and |Φ〉 are

12 $$p(\Psi )=[\alpha ,\,(1-\alpha ){b}_{1},\,(1-\alpha ){b}_{2},\,(1-\alpha )(1-{b}_{1}-{b}_{2})],$$

13 $$p(\Phi )=[\beta c,\,\beta (1-c),\,(1-\beta )d,\,(1-\beta )(1-d)].$$

In addition, we assume that $\alpha \ge 1/2$, which implies that *p*(Ψ) is already sorted in a decreasing order. Regarding the vector *p*(Φ), there are in principle six different ways of sorting its components. However, we only consider the case in which *p*(Φ) is already sorted. This restriction is equivalent to consider $\beta \ge \frac{d}{1-c+d}$.

Let us recall that $|\Psi \rangle \mathop{\to }\limits_{{\rm{IO}}}|\Phi \rangle$ if and only if $p(\Phi )\,\succcurlyeq \,p(\Psi )$, which in this case implies

14 $$\beta c\ge \alpha ,\,\beta \ge \alpha +(1-\alpha ){b}_{1},\,\,\,{\rm{a}}{\rm{n}}{\rm{d}}\,\,\,\beta +(1-\beta )d\ge \alpha +(1-\alpha ){b}_{1}+(1-\alpha ){b}_{2}.$$

Under these constraints we obtain the following proposition:

### Proposition 1.

*Let* $|a\rangle$, $|b\rangle$, $|c\rangle$, $|d\rangle$ *be pure states as in* Eqs. (5)–(8) *that satisfy the conditions* (2) *and* (3). *Let* $|\Psi \rangle =\sqrt{\alpha }|a\rangle +\sqrt{1-\alpha }|b\rangle$ *and* $|\Phi \rangle =\sqrt{\beta }|c\rangle +\sqrt{1-\beta }|d\rangle$ *be arbitrary superpositions*, *such that* $\alpha \ge \frac{1}{2}$ *and* $\beta \ge {\mathop{\beta }\limits^{ \sim }}_{0}\equiv \frac{d}{1-c+d}$. *In addition*, *let us define* ${\tilde{\beta }}_{1}\equiv \frac{1}{2c}$, ${\tilde{\beta }}_{2}\equiv \frac{1+{b}_{1}}{2}$, ${\tilde{\beta }}_{3}\equiv \frac{1+{b}_{1}+{b}_{2}-2d}{2(1-d)}$, ${\tilde{\beta }}_{4}\equiv \frac{{b}_{1}}{1-c+{b}_{1}c}$, *and* ${\tilde{\beta }}_{5}\equiv \frac{{b}_{1}+{b}_{2}-d}{1-d-c(1-{b}_{1}-{b}_{2})}$.

*Taking into account the previous scenario*, *we have the following results*:

1. *If* $${\tilde{\beta }}_{1}\ge \,{\rm{\max }}\,\{{\tilde{\beta }}_{2},{\tilde{\beta }}_{3}\}$$, *then*
  15 $$|\Psi \rangle \,\mathop{\to }\limits_{{\rm{I}}{\rm{O}}}|\,\Phi \rangle \,\,\,\,\Longleftrightarrow \,\,\,\,max\,\{{\mathop{\beta }\limits^{ \sim }}_{0},{\mathop{\beta }\limits^{ \sim }}_{1}\}\le \beta  < 1,\,\,\frac{1}{2}\le \alpha \le \beta c.$$
2. *If* $${\tilde{\beta }}_{2}\ge \,{\rm{\max }}\,\{{\tilde{\beta }}_{1},{\tilde{\beta }}_{3}\}$$, *then*
  16 $$|\Psi \rangle \mathop{\to }\limits_{{\rm{I}}{\rm{O}}}|\Phi \rangle \,\,\,\,\Longleftrightarrow \,\,\,\,\{\begin{array}{cc}max\,\{{\mathop{\beta }\limits^{ \sim }}_{0},{\mathop{\beta }\limits^{ \sim }}_{2}\}\le \beta \le {\mathop{\beta }\limits^{ \sim }}_{4}, & \frac{1}{2}\le \alpha \le \frac{\beta -{b}_{1}}{1-{b}_{1}},\,\,{\rm{o}}{\rm{r}}\\ max\,\{{\mathop{\beta }\limits^{ \sim }}_{0},{\mathop{\beta }\limits^{ \sim }}_{4}\}\le \beta \le 1, & \frac{1}{2}\le \alpha \le \beta c.\end{array}$$
3. *If* $${\tilde{\beta }}_{3}\ge \,{\rm{\max }}\,\{{\tilde{\beta }}_{1},{\tilde{\beta }}_{2}\}$$, *then*

17 $$|\Psi \rangle \mathop{\to }\limits_{{\rm{I}}{\rm{O}}}|\Phi \rangle \,\,\,\,\Longleftrightarrow \,\,\,\,\{\begin{array}{cc}max\,\{{\mathop{\beta }\limits^{ \sim }}_{0},{\mathop{\beta }\limits^{ \sim }}_{3}\}\le \beta \le {\mathop{\beta }\limits^{ \sim }}_{5}, & \frac{1}{2}\le \alpha \le \frac{\beta (1-d)+d-{b}_{1}-{b}_{2}}{1-{b}_{1}-{b}_{2}},\,\,{\rm{o}}{\rm{r}}\\ max\,\{{\mathop{\beta }\limits^{ \sim }}_{0},{\mathop{\beta }\limits^{ \sim }}_{5}\}\le \beta \le 1, & \frac{1}{2}\le \alpha \le \beta c.\end{array}$$

**Case:** $$(2,2)\mathop{\to }\limits_{{\rm{IO}}}(2,2)$$

In this case, the coherent states $|a\rangle$, $|b\rangle$, $|c\rangle$, $|d\rangle$ have the following form

18 $$|a\rangle =\sqrt{a}|0\rangle +\sqrt{1-a}|1\rangle ,$$

19 $$|b\rangle =\sqrt{b}|2\rangle +\sqrt{1-b}|3\rangle ,$$

20 $$|c\rangle =\sqrt{c}|0\rangle +\sqrt{1-c}|1\rangle ,$$

21 $$|d\rangle =\sqrt{d}|2\rangle +\sqrt{1-d}|3\rangle ,$$

with $a\ge b > 0$, $c\ge d > 0$ and $a,\,b,\,c,\,d\ge 1/2$. As in the previous case, we can sort the coefficients in this way without loss of generality.

In order to satisfy the conditions (2) and (3), the coefficients $a,\,b,\,c,\,d$ have to satisfy the following constraints: $a > \,{\rm{\max }}\{c,d\}$ and $b > \,{\rm{\max }}\{c,d\}$. Therefore, the coefficients have to satisfy

22 $$a\ge b > c\ge d\ge 1-d\ge 1-c > 1-b\ge 1-a > 0.$$

In this case, the initial and final superpositions have the following form

23 $$|\Psi \rangle =\sqrt{\alpha a}|0\rangle +\sqrt{\alpha (1-a)}|1\rangle +\sqrt{(1-\alpha )b}|2\rangle +\sqrt{(1-\alpha )(1-b)}|3\rangle ,$$

and

24 $$|\Phi \rangle =\sqrt{\beta c}|0\rangle +\sqrt{\beta (1-c)}|1\rangle +\sqrt{(1-\beta )d}|2\rangle +\sqrt{(1-\beta )(1-d)}|3\rangle .$$

Therefore, the probability vectors in the incoherent basis associated with the superpositions |Ψ〉 and |Φ〉 are

25 $$p(\Psi )=[\alpha a,\,\alpha (1-a),\,(1-\alpha )b,\,(1-\alpha )(1-b)],$$

26 $$p(\Phi )=[\beta c,\,\beta (1-c),\,(1-\beta )d,\,(1-\beta )(1-d)].$$

In addition, we assume that *p*(Ψ) and *p*(Φ) are already sorted in a decreasing order, which is equivalent to assume that $\alpha (1-a)\ge (1-\alpha )b$ and $\beta (1-c)\ge (1-\beta )d$. In this case, the majorization condition $p(\Phi )\,\succcurlyeq \,p(\Psi )$ implies

27 $$\beta c\ge \alpha a,\,\,\,\beta \ge \alpha ,\,\,\,{\rm{a}}{\rm{n}}{\rm{d}}\,\,\,\beta +(1-\beta )d\ge \alpha +(1-\alpha )b.$$

Under these constraints we obtain the following proposition:

### Proposition 2.

*Let* $|a\rangle$, $|b\rangle$, $|c\rangle$, $|d\rangle$ *be coherent states as in* Eqs. (18)–(21) *that satisfy the conditions* (2) *and* (3). *Let* $|\Psi \rangle =\sqrt{\alpha }|a\rangle +\sqrt{1-\alpha }|b\rangle$ *and* $|\Phi \rangle =\sqrt{\beta }|c\rangle +\sqrt{1-\beta }|d\rangle$ *be arbitrary superpositions*, *such that* $\alpha \ge {\tilde{\alpha }}_{0}\equiv \frac{b}{1-a+b}$ *and* $\beta \ge {\mathop{\beta }\limits^{ \sim }}_{0}\equiv \frac{d}{1-c+d}$. *In addition*, *let us define* ${\tilde{\beta }}_{1}\equiv \frac{ab}{c(1-a+b)}$, ${\tilde{\beta }}_{2}\equiv \frac{b(2-a)-d(1-a+b)}{(1-d)(1-a+b)}$ *and* ${\tilde{\beta }}_{3}\equiv \frac{a(d-b)}{c(1-b)+a(d-1)}$.

*With respect to the scenario described above*, *we obtain the following results*:

1. *If* $${\tilde{\beta }}_{1}\ge {\tilde{\beta }}_{2}$$, *then*
  28 $$|\Psi \rangle \mathop{\to }\limits_{{\rm{I}}{\rm{O}}}|\Phi \rangle \,\,\,\,\Longleftrightarrow \,\,\,\,max\,\{{\mathop{\beta }\limits^{ \sim }}_{0},{\mathop{\beta }\limits^{ \sim }}_{1}\}\le \beta  < 1,\,\,{\mathop{\alpha }\limits^{ \sim }}_{0}\le \alpha \le \frac{\beta c}{a}.$$
2. *If* $${\tilde{\beta }}_{2} > {\tilde{\beta }}_{1}$$, *then*

29 $$|\Psi \rangle \mathop{\to }\limits_{{\rm{I}}{\rm{O}}}|\Phi \rangle \,\,\,\,\Longleftrightarrow \,\,\,\,\{\begin{array}{cc}max\,\{{\mathop{\beta }\limits^{ \sim }}_{0},{\mathop{\beta }\limits^{ \sim }}_{2}\}\le \beta \le {\mathop{\beta }\limits^{ \sim }}_{3}, & {\mathop{\alpha }\limits^{ \sim }}_{0}\le \alpha \le \frac{\beta (1-d)+d-b}{1-b},\,\,{\rm{o}}{\rm{r}}\\ max\,\{{\mathop{\beta }\limits^{ \sim }}_{0},{\mathop{\beta }\limits^{ \sim }}_{3}\}\le \beta \le 1, & {\mathop{\alpha }\limits^{ \sim }}_{0}\le \alpha \le \frac{\beta c}{a}.\end{array}$$

### Comparison with the relative entropy of coherence criterion

A necessary condition for the transformation $|\Psi \rangle \mathop{\to }\limits_{{\rm{IO}}}|\Phi \rangle$ can be obtained using the relative entropy of coherence criterion:

30 $$|\Psi \rangle \mathop{\to }\limits_{{\rm{I}}{\rm{O}}}|\Phi \rangle \,\,\,\Longrightarrow \,\,\,{C}_{{\rm{r}}{\rm{e}}}(|\Psi \rangle )\ge {C}_{{\rm{r}}{\rm{e}}}(|\Phi \rangle ),$$

with ${C}_{{\rm{re}}}(\rho )$ the relative entropy of coherence, which in the case of pure states reduces to

31 $${C}_{{\rm{re}}}(|\Psi \rangle )=H(p(\Psi )),$$

where $H(p(\Psi ))=-\,{\sum }_{i}\,{p}_{i}(\Psi )\,\log \,p{}_{i}(\Psi )$ is the Shannon entropy of the probability vector associated with the state |Ψ〉 in the incoherent basis. More generally, we can use any coherence monotone measure to formulate alternative necessary criteria (see, *e*.*g*., ref. ^3^).

**Figure 1:**
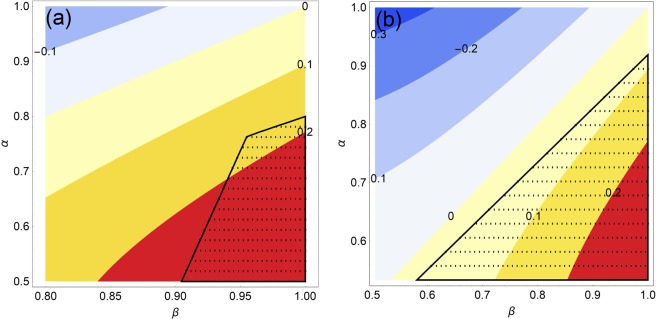
In Figure (**1a**) pure states given in Eqs. (5–8) (with ${b}_{1}=0.809073$, ${b}_{2}=0.114367$, $c=0.8$ and $d=0.79822$) are considered. The contour plot shows the difference between the relative entropy of coherence of the initial and final superpositions, ${C}_{{\rm{re}}}(|\Psi \rangle )-{C}_{{\rm{re}}}(|\Phi \rangle )$, for $0.8\le \alpha \le 1$ and $0.5\le \beta \le 1$. The dotted figure represents the values of $\alpha$ and $\beta$ for which the transition $|\Psi \rangle \mathop{\to }\limits_{{\rm{IO}}}|\Phi \rangle$ is allowed, according to Proposition 1. In Figure (**1b**) pure states given in Eqs. (18–21) (with $a=0.55058$, $b=0.514271$, $c=0.506075$ and $d=0.503128$) are considered. Again, the contour plot shows the difference between the relative entropy of coherence of the initial and final superpositions, for $0.5\le \alpha ,\,\beta \le 1$. The dashed black figure represents the values of $\alpha$ and $\beta$ for which the transition $|\Psi \rangle \mathop{\to }\limits_{{\rm{IO}}}|\Phi \rangle$ is allowed, according to Proposition 2.

In Fig. 1(a), we consider particular pure states $|a\rangle$, $|b\rangle$, $|c\rangle$ and $|d\rangle$ as in Eqs. (5–8), with ${b}_{1}=0.809073$, ${b}_{2}=0.114367$, $c=0.8$ and $d=0.79822$. These quantum states satisfy the inequalities given in (9). For this case, we plot the difference between the relative entropy of coherence of the superpositions |Ψ〉 and |Φ〉, i.e., ${C}_{{\rm{re}}}(|\Psi \rangle )-{C}_{{\rm{re}}}(|\Phi \rangle )$, for $0.8\le \beta \le 1$ and $0.5\le \alpha \le 1$. In addition, we plot the region of $\alpha$ and $\beta$ where the transition $|\Psi \rangle \mathop{\to }\limits_{{\rm{IO}}}|\Phi \rangle$ is allowed, according to the results of Proposition 1. In Fig. 1(b), we consider another set of pure states $|a\rangle$, $|b\rangle$, $|c\rangle$ and $|d\rangle$ as in Eqs. (18–21), with $a=0.55058$, $b=0.514271$, $c=0.506075$ and $d=0.503128$. These states satisfy the inequalities given in (22). Again, we plot the difference between the relative entropy of coherence of the states |Ψ〉 and |Φ〉, for $0.5\le \beta ,\,\alpha \le 1$. Finally, we plot the region of $\alpha$ and $\beta$ where the transition under consideration is possible, according to the results of Proposition 2. Both examples show that the criterion based on the relative entropy of coherence measure does not provide a sufficient condition for the transformation $|\Psi \rangle \mathop{\to }\limits_{{\rm{IO}}}|\Phi \rangle$.

## Discussion

Quantum coherence is not only a fundamental notion of quantum mechanics, but also a useful quantum resource used in quantum information processing. Since coherence is a consequence of the superposition principle, it is relevant to understand how the coherence of a superposition of coherent states is related with the coherence of the superposed states.

In this work, we have considered two pure initial states and two pure final coherent states, such that the former ones cannot be transformed into the latter ones by means of incoherent transformations. In this situation, we have analyzed the conditions for the existence of superpositions of the initial states that can be transformed into superpositions of the final states. In particular, we have considered superpositions of quantum states belonging to orthogonal subspaces. By appealing to the majorization theory, we have obtained the necessary and sufficient conditions so that the transformations under consideration are possible.

For the initial superposition state we have considered two cases: (1) The initial states is a superposition of two states, each one with two non-null coefficients in the fixed basis. (2) The initial states is a superposition of two states, with one and three non-null coefficients in the fixed basis, respectively. For the final state, we have considered a superposition of two coherent states, each one with two non-null coefficients in the fixed basis. In Propositions 1 and 2, we have obtained necessary and sufficient conditions for such transformations to be possible.

Finally, we have provided two examples that illustrate the difference between the conditions obtained in Propositions 1 and 2, and the necessary criterion based on the relative entropy of coherence.

## Methods

*Proof of Proposition* 1:

From the majorization condition (1) and some algebra, we have

32 $$|\Psi \rangle \mathop{\to }\limits_{{\rm{I}}{\rm{O}}}|\Phi \rangle \,\,\,\,\Longleftrightarrow \,\,\,\,\alpha \le \beta c,\,\,\alpha \le \frac{\beta -{b}_{1}}{1-{b}_{1}}\,\,{\rm{a}}{\rm{n}}{\rm{d}}\,\,\alpha \le \frac{\beta (1-d)+d-{b}_{1}-{b}_{2}}{1-{b}_{1}-{b}_{2}}.$$

Notice that ${\tilde{\beta }}_{1}$, ${\tilde{\beta }}_{2}$, ${\tilde{\beta }}_{3}$, ${\tilde{\beta }}_{4}$ and ${\tilde{\beta }}_{5}$ are the values of $\beta$ that satisfy the equations $\frac{1}{2}=\beta c$, $\frac{1}{2}=\frac{\beta -{b}_{1}}{1-{b}_{1}}$, $\frac{1}{2}=\frac{\beta (1-d)+d-{b}_{1}-{b}_{2}}{1-{b}_{1}-{b}_{2}}$, $\beta c=\frac{\beta -{b}_{1}}{1-{b}_{1}}$ and $\beta c=\frac{\beta (1-d)+d-{b}_{1}-{b}_{2}}{1-{b}_{1}-{b}_{2}}$, respectively.

1. Let us assume ${\tilde{\beta }}_{1}\ge {\tilde{\beta }}_{2}$ and ${\tilde{\beta }}_{1}\ge {\tilde{\beta }}_{3}$. Then, $\beta c\le \frac{\beta -{b}_{1}}{1-{b}_{1}}$ and $\beta c\le \frac{\beta (1-d)+d-{b}_{1}-{b}_{2}}{1-{b}_{1}-{b}_{2}}$ for ${\tilde{\beta }}_{1}\le \beta \le 1$. Taking into account the additional conditions $\frac{1}{2}\le \alpha$ and ${\tilde{\beta }}_{0}\le \beta$, we obtain that (32) is equivalent to (15).
2. Let us assume ${\tilde{\beta }}_{2}\ge {\tilde{\beta }}_{1}$ and ${\tilde{\beta }}_{2}\ge {\tilde{\beta }}_{3}$. The last condition implies $\frac{\beta -{b}_{1}}{1-{b}_{1}}\le \frac{\beta \mathrm{(1}-d)+d-{b}_{1}-{b}_{2}}{1-{b}_{1}-{b}_{2}}$ for $0\le \beta \le 1$. Let us compare the expressions $\beta c$ and $\frac{\beta -{b}_{1}}{1-{b}_{1}}$ in the range ${\tilde{\beta }}_{2}\le \beta \le 1$. It can be shown that $\frac{\beta -{b}_{1}}{1-{b}_{1}}\le \beta c$ for ${\tilde{\beta }}_{2}\le \beta \le {\tilde{\beta }}_{4}$, whereas $\beta c\le \frac{\beta -{b}_{1}}{1-{b}_{1}}$ for ${\tilde{\beta }}_{4}\le \beta \le 1$. Taking into account the additional conditions $\frac{1}{2}\le \alpha$ and ${\tilde{\beta }}_{0}\le \beta$, we obtain that (32) is equivalent to (16).
3. Let us assume ${\tilde{\beta }}_{3}\ge {\tilde{\beta }}_{1}$ and ${\tilde{\beta }}_{3}\ge {\tilde{\beta }}_{2}$. The last condition implies $\frac{\beta \mathrm{(1}-d)+d-{b}_{1}-{b}_{2}}{1-{b}_{1}-{b}_{2}}\le \frac{\beta -{b}_{1}}{1-{b}_{1}}$ for $0\le \beta \le 1$. Let us compare the expressions $\beta c$ and $\frac{\beta \mathrm{(1}-d)+d-{b}_{1}-{b}_{2}}{1-{b}_{1}-{b}_{2}}$ in the region ${\tilde{\beta }}_{3}\le \beta \le 1$. It can be shown that $\frac{\beta \mathrm{(1}-d)+d-{b}_{1}-{b}_{2}}{1-{b}_{1}-{b}_{2}}\le \beta c$ for ${\tilde{\beta }}_{3}\le \beta \le {\tilde{\beta }}_{5}$, whereas $\beta c\le \frac{\beta \mathrm{(1}-d)+d-{b}_{1}-{b}_{2}}{1-{b}_{1}-{b}_{2}}$ for ${\tilde{\beta }}_{5}\le \beta \le 1$. Taking into account the additional conditions $\frac{1}{2}\le \alpha$ and ${\tilde{\beta }}_{0}\le \beta$, we obtain that (32) is equivalent to (17). □

*Proof of Proposition* 2:

From the majorization condition (1) and some algebra, we have

33 $$|\Psi \rangle \mathop{\to }\limits_{{\rm{I}}{\rm{O}}}|\Phi \rangle \,\,\,\,\Longleftrightarrow \,\,\,\,\alpha \le \frac{\beta c}{a},\,\,\alpha \le \beta \,\,{\rm{a}}{\rm{n}}{\rm{d}}\,\,\alpha \le \frac{\beta (1-d)+d-b}{1-b}.$$

Notice that $\frac{\beta c}{a} < \beta$ for $0\le \beta \le 1$. In addition, notice that ${\tilde{\beta }}_{1}$, ${\tilde{\beta }}_{2}$ and ${\tilde{\beta }}_{3}$ are the values of $\beta$ that satisfy the equations ${\tilde{\alpha }}_{0}=\frac{\beta c}{a}$, ${\tilde{\alpha }}_{0}=\frac{\beta (1-d)+d-b}{1-b}$ and $\frac{\beta c}{a}=\frac{\beta (1-d)+d-b}{1-b}$, respectively.

1. Let us assume ${\tilde{\beta }}_{1}\ge {\tilde{\beta }}_{2}$. It can be shown that $\frac{\beta c}{a}\le \frac{\beta \mathrm{(1}-d)+d-b}{1-b}$ for ${\tilde{\beta }}_{1}\le \beta \le 1$. Taking into account the additional conditions ${\tilde{\alpha }}_{0}\le \alpha$ and ${\tilde{\beta }}_{0}\le \beta$, we obtain that (33) is equivalent to (28).
2. Let us assume ${\tilde{\beta }}_{1} < {\tilde{\beta }}_{2}$. It can be shown that $\frac{\beta \mathrm{(1}-d)+d-b}{1-b}\le \frac{\beta c}{a}$ for ${\tilde{\beta }}_{2}\le \beta \le {\tilde{\beta }}_{3}$, whereas $\frac{\beta c}{a}\le \frac{\beta \mathrm{(1}-d)+d-b}{1-b}$ for ${\tilde{\beta }}_{3}\le \beta \le 1$. Taking into account the additional conditions ${\tilde{\alpha }}_{0}\le \alpha$ and ${\tilde{\beta }}_{0}\le \beta$, we obtain that (33) is equivalent to (29).□
